# Decoupling T cell cytotoxicity: a CCL21+ICAM1-based synthetic immune niche enhances tumor elimination by accelerating lytic hit delivery

**DOI:** 10.1186/s12885-026-16748-y

**Published:** 2026-10-15

**Authors:** Manish Kumar Gupta, Rawan Zoabi, Sofi Yado, Jörn Starruß, Benjamin Geiger, Haralampos Hatzikirou

**Affiliations:** 1 Center for Interdisciplinary Digital Sciences, Department Information Services and High Performance Computing, TUD Dresden University of Technology, Dresden, Germany; 2 Department of Immunology and Regenerative Biology, Weizmann Institute of Science, Rehovot, Israel; 3 Department of Mathematics, Khalifa University, Abu Dhabi, UAE; 4 Health and Life Sciences, European University Cyprus at Frankfurt, Frankfurt, Germany

**Keywords:** CD8$^+$ T cell, Cytotoxicity, Parameter estimation, Practical identifiability, Sensitivity analysis, Multi-hit theory

## Abstract

Preserving T cell cytotoxic function during ex vivo expansion remains a major challenge for adoptive cancer immunotherapy. A synthetic immune niche (SIN) composed of immobilized CCL21 and ICAM1 was shown to improve T cell expansion while preserving cytotoxicity; however, it remains unclear which specific step of the T cell killing process is enhanced by the SIN stimulation. Here, we combined advanced imaging based on time-lapse microscopy with a mean-field model to analyze the distinct stages in killing of B16 melanoma cells by CD8$^+$ T cells. This framework enabled us to resolve the tumor cell killing process into discrete steps throughout target cell engagement with SIN-treated T cells and lytic hit delivery. We found that tumor cell death is best explained by a multi-hit process, requiring approximately four discrete hits to trigger cell death. Model-based analysis identified a significant increase in the lytic hit-delivery rate, $k_3$, as the main kinetic change associated with the enhanced cytotoxicity of SIN-treated T cells. The target encounter rate, $k_1$, could not be reliably compared between conditions because it was practically non-identifiable in the untreated dataset. Global sensitivity analysis further showed that increasing lytic hit delivery has a stronger effect on tumour control than increasing target encounter rates. These findings fundamentally reorient our understanding of optimized T cell manufacturing, suggesting that the lytic execution step may be the primary rate-limiting bottleneck in this in-vitro system, and that engineering strategies targeting granule polarization or discharge warrant prioritization alongside affinity-enhancement approaches.

**Supplementary Information:**

The online version contains supplementary material available at https://doi.org/10.1186/s12885-026-16748-y.

## Introduction

Adoptive cancer immunotherapy by tumor-infiltrating lymphocyte (TIL) and chimeric antigen receptor (CAR) T has demonstrated remarkable efficacy in refractory malignancies [1, 2], yet realizing its full potential remains a major challenge. A central manufacturing bottleneck is the *ex vivo* expansion of patient-derived T cells to clinically relevant numbers while preserving functional potency [3]. Standard protocols rely on artificial APC surrogates such as anti-CD3/CD28-coated microbeads, which provide strong mitogenic stimulation but lack the structural and soluble complexity of the native lymphoid niche. As a result, T cells expanded under these conditions frequently differentiate toward terminal effector states and acquire features of exhaustion that compromise in vivo persistence and cytotoxic function [4–7]. This proliferation–differentiation trade-off remains a pervasive obstacle in the field.

To address this limitation, a synthetic immune niche (SIN) was developed, which recreates, *ex vivo*, key stimulatory features of the lymphoid and the tumor-associated microenvironments [8]. Substrates co-functionalized with the chemokine CCL21 and the integrin ligand ICAM1 support a robust T cell expansion while preserving, and in some cases augmenting, effector function [9, 10]. While the phenomenological outcomes of SIN treatment, namely, high yield of T cells with high killing capacity, are promising, the biological processes driving this enhanced cytotoxicity remain poorly understood. Enhanced killing can, in principle, be attributed to three distinct intercellular processes: An increased frequency of T cell–tumor cell encounters, prolonged contact duration, or a more efficient lytic hit delivery, once contact is established. Which of these processes SIN conditioning actually modulates — and to what degree — remains an open question with direct implications for therapeutic design. Distinguishing between these possibilities is challenging using standard population-level assays (e.g., Chromium-51 release or flow cytometry), which integrate these processes into a single endpoint measurement.

Mathematical modeling has long been used to study T cell cytotoxicity, but existing frameworks differ substantially in the degree to which they resolve the underlying biology. Early models of tumor–cytotoxic T lymphocyte (CTL) interactions typically used predator–prey or mass-action formulations, in which tumor cells proliferate and are eliminated through a single effective killing term proportional to effector and target abundance [11, 12]. While such models capture bulk killing kinetics, they do not distinguish between the elementary steps that generate cytotoxicity, such as target encounter, contact persistence, and productive lytic hit delivery. In contrast, stochastic and agent-based models, monitored by live-cell imaging, have shown that cytotoxicity is a multistep and heterogeneous process involving cell migration, conjugate formation, synapse maintenance, and serial or cooperative killing [13–15]. These studies, together with single-cell experiments, further suggest that tumor cell death is often a complex, rather than binary process, and that may require repeated or additive sublethal hits rather than a single lethal event [16–18]. However, although these richer models provide a detailed mechanistic view of the killing process, calibrating them from population-level measurements is often challenging because their larger parameter spaces can limit identifiability.

**Fig. 1:**
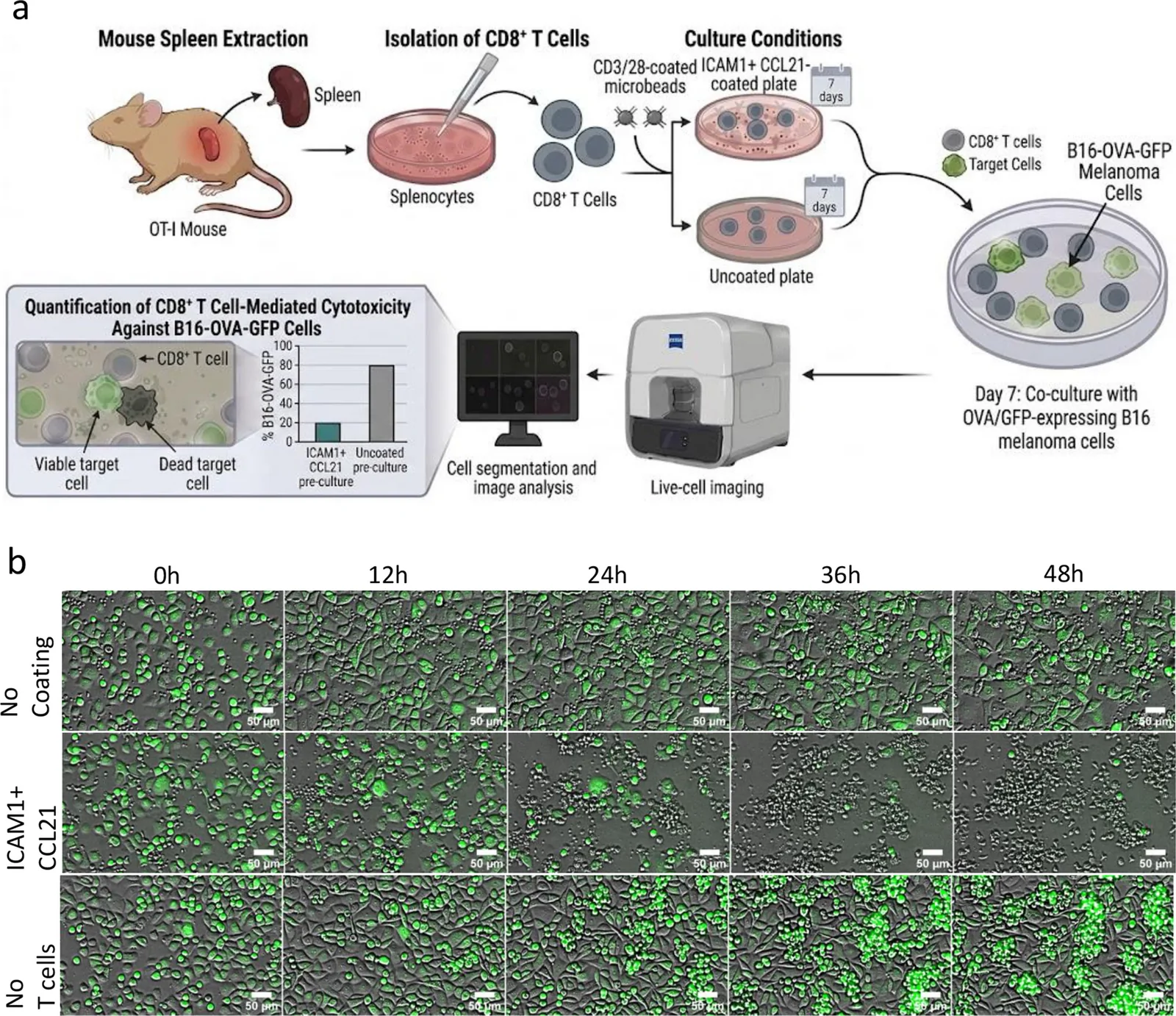
Schematic of the experimental workflow. **a** CD8$^{+}$ T cells were isolated from spleens of OT-I mice, activated with CD3/28-coated microbeads, and cultured for seven days on either ICAM1 + CCL21-coated or uncoated surfaces. On day 7, SIN-treated and untreated T cells were co-cultured with antigen (OVA)- and GFP-expressing B16 melanoma cells. Live-cell imaging was performed using a Celldiscoverer 7 microscope, followed by cell segmentation and image analysis to quantify T-cell cytotoxicity, measured as loss of GFP fluorescence in target cells. **b** Representative transmitted-light and fluorescence time-lapse images of target cells co-cultured with control (untreated) T cells (top, Supplementary movie 1), SIN-treated T cells (middle, Supplementary movie 2), or without effector cells (bottom, Supplementary movie 3). Live target cells are GFP$^{+}$ (green); T cells are unlabeled. Scale bar, 50 µm. Images are representative of 4 independent experiments, each performed in technical duplicate

We therefore developed a coarse-grained mean-field model to address this biological question while retaining the key multistep structure. In this framework, target encounter is represented by an effective binding rate ($k_1$), conjugate stability by a dissociation rate ($k_2$), and productive killing by a lytic hit delivery rate ($k_3$). By fitting the model to high-throughput live-cell imaging data of OT-I CD8$^+$ T cells interacting with OVA/GFP-expressing B16 melanoma cells over 48 h (Fig. 1), we investigated which of these effective kinetic steps differs between control and SIN-expanded T cells. We found that SIN-stimulated T cells show a marked increase in lytic hit delivery rate, a result we term “synaptic lethality.” Global sensitivity analysis further confirms that $k_3$ exerts dominant control over tumor population dynamics, suggesting that future therapeutic optimization should prioritize the quality of the lytic event over the frequency or duration of cellular contacts.

## Results

### A multi-hit framework accurately describes tumor elimination dynamics

To rigorously investigate the cytotoxic interplay between T cells and tumor cells, we constructed a mean-field mathematical model that moves beyond simple mass-action kinetics to incorporate the concept of cumulative damage. Experimental observations of the co-culture system, OT-I T cells and B16-OVA-GFP melanoma cells, revealed a nonlinear decay in the tumor cell population. Specifically, we observed an initial “shoulder” phase, representing a delay in the onset of significant tumor cell death, which is characteristic of systems where a threshold of damage must be exceeded before an irreversible state transition occurs.

We formulated the interaction as a system of coupled ordinary differential equations (ODEs) describing the transition of tumor cells through a series of discrete damage states, denoted by $C_i$, where *i* represents the number of accumulated lethal “hits” (*i = 0, 1, … , n*). The model incorporates four fundamental biological processes:

1. Tumor growth: Viable undamaged tumor cells proliferate logistically with a specific growth rate *μ* and a carrying capacity *K*. We assume that partially damaged cells (*i>0*) are arrested in the cell cycle, thus not contributing to proliferation.
2. Conjugation (Binding): Free T cells (*T*) bind to tumor cells in any state $C_i$ with a second-order association rate constant $k_1$, forming a physical complex representing the “search and capture” phase of cytotoxicity.
3. Complex dissociation (Unbinding): T cells dissociate from the complex with a first-order dissociation rate $k_2$, returning the tumor cell to state $C_i$ and the T cell to the free pool. This reversibility is crucial, as it allows T cells to detach and engage new targets (serial killing).
4. Hit delivery (Lethal step): Within the conjugated complex, the T cell executes a lytic event (e.g., granule exocytosis) with a first-order rate constant $k_3$. This transition moves the tumor cell from damage state $C_i$ to $C_{i+1}$, effectively advancing it toward apoptosis.

**Fig. 2:**
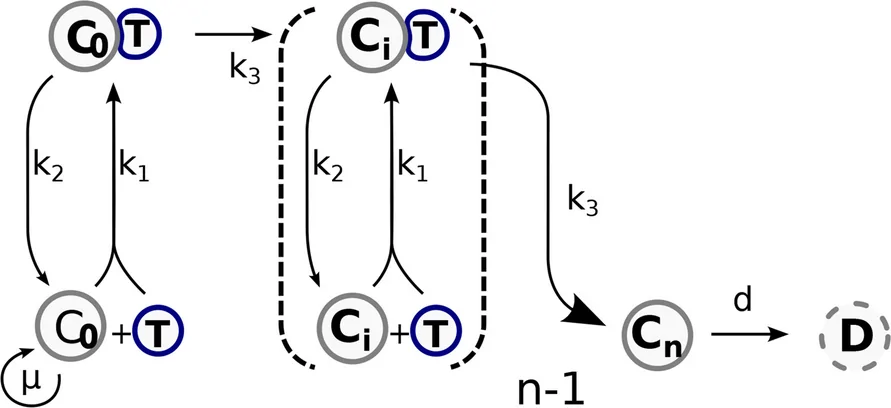
Schematic of the multi-hit cytotoxicity model. Tumor cells progress from the undamaged state $C_0$ through successive damage states $C_i$ upon T-cell-mediated lytic hit delivery. Free T cells bind targets with rate $k_1$, dissociate with rate $k_2$, and deliver hits within conjugates at rate $k_3$. After *n* accumulated hits, target cells enter the apoptotic state $C_n$ and decay with rate *d*

The final state $C_n$ represents the irreversibly committed apoptotic cell, which subsequently decays (loses membrane integrity and fluorescence) at a rate *d*. The model structure is illustrated schematically in Fig. 2, while the corresponding system of ordinary differential equations is provided in the STAR Methods (Multi-hit ODE model).

The model further assumes that sublethal damage is not repaired over the 48 h experimental timescale, such that tumor cells progress irreversibly through the discrete damage states. This approximation is motivated by the high-affinity OT-I/B16-OVA system, in which cytotoxic damage is expected to accumulate on experimentally relevant timescales. For simplicity, we also neglect T-cell proliferation and exhaustion during the assay, and treat the free T-cell evolving only through binding and unbinding, while the total T-cell remains temporally constant.

### In vitro B16 melanoma cells require approximately four hits to commit to apoptosis

A central question in T cell biology concerns the quantification of lethality: is a single degranulation event sufficient to induce target-cell death? To address this question, we treated the number of hits, *n*, as a key structural parameter. We fitted the model to time-series measurements of tumour population size under both SIN-treated and untreated conditions, systematically varying *n* from 1 (single-hit) to 9.

Model performance was evaluated using two metrics: the mean squared error (MSE), which quantifies the deviation between the simulations and the data, and the Akaike information criterion (AIC) [19], which penalizes model complexity and guards against overfitting. As expected, increasing *n* introduced a longer delay, or shoulder, in the tumour decay curve, thereby improving the fit to the early time points. The MSE decreased sharply as *n* increased from 1 to 3 and then reached a plateau. This indicates that single-hit kinetics (*n=1*) are insufficient to capture the observed delay in B16 apoptosis.

**Fig. 3:**
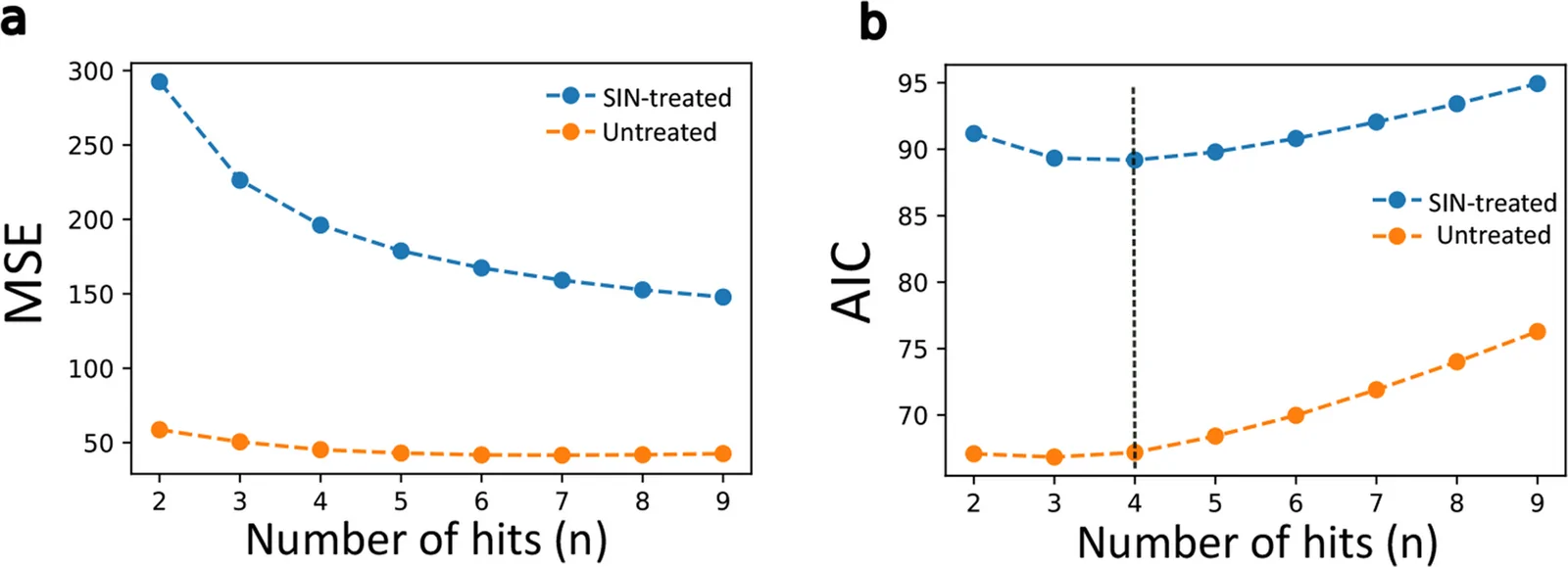
Model selection: **a** Mean squared error (MSE) obtained from model fitting as a function of the number of hits, *n*, for SIN-treated and untreated conditions. **b** Akaike information criterion (AIC) as a function of *n*. Note: The AIC minima occurred at (*n=4*) for the SIN-treated condition and (*n=3*) for the untreated condition. A consensus value of (*n=4*), indicated by the vertical dashed line, was used for both conditions

The AIC provided a more distinct criterion for model selection. For the SIN-treated condition, the AIC reached a minimum at *n=4*. For the untreated condition, the minimum was observed at *n=3*, with *n=4* yielding a very similar value. To maintain structural consistency and enable direct parameter comparisons between conditions, we selected *n=4* as the consensus hit number for the remainder of the analysis. This result suggests that, within the model framework, B16 melanoma cells behave as signal integrators and require the accumulation of approximately four cytotoxic hit events before committing irreversibly to cell death (Fig. 3).

### Direct experimental estimation constrains the parameter space

One of the central challenges in Systems Biology is parameter identifiability—the ability to uniquely determine parameter values from observed data. To mitigate this, we employed a “divide and conquer” strategy, estimating a subset of model parameters directly from image-derived measurements and then fitting only the remaining parameters using the ordinary differential equation (ODE) model, thereby reducing the number of free parameters.

#### Decay rate (*d*):

**Fig. 4:**
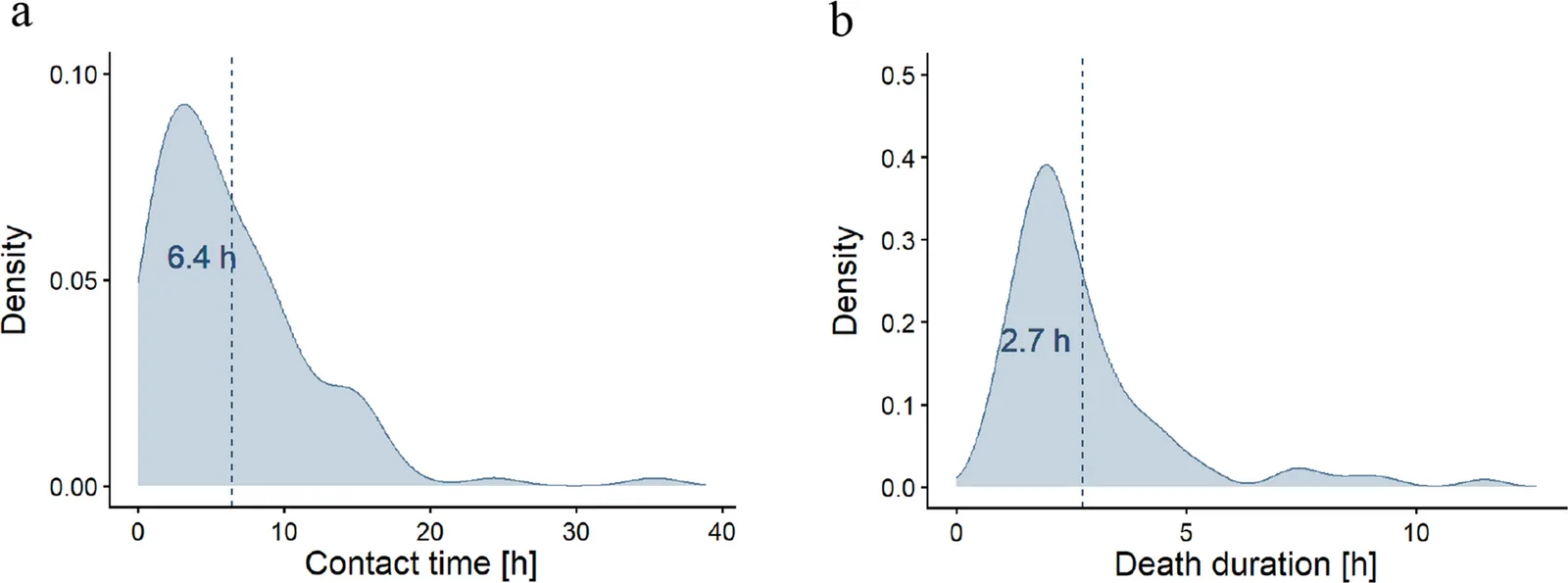
Experimental estimate of T cell-target cell dissociation rate $(k_2)$ and cell death duration. Density plots of the contact time between T cells and target cells (**a**) and the interval between target cell rounding up and the loss of GFP (cell death duration; **b**), based on combined data from SIN-treated and untreated conditions. Vertical lines indicate the mean

We quantified the duration of the cell death phase as the time interval between cell rounding (morphological commitment) and the complete loss of GFP fluorescence (membrane permeabilization/death), with this combination serving as a proxy for target cell death. Tracking individual cell-death events yielded a distribution of decay time durations with a population mean $T_{\textrm{avg}} \approx 164~\textrm{min}$ (Fig. 4b). Assuming a first-order (exponential) execution phase, the decay rate was estimated as $d = \frac{1}{T_{\textrm{avg}}} \approx 0.006~\textrm{min}^{-1}$. This value was consistent across experimental conditions, suggesting that the execution phase of death is intrinsic to the tumor cell and is not statistically significantly altered by the T cell’s activation history (SI Fig. 2).

#### Dissociation rate ($k_2$):

Similarly, we measured the duration of physical conjugation events between T cells and tumor cells (Fig. 4a). We assumed that T cell–tumor cell dissociation follows a first-order stochastic process with constant rate $k_2$. Based on the measured mean attachment duration $\langle T_{\textrm{attach}} \rangle \approx 400~\textrm{min}$; the dissociation rate was estimated as $k_2 = \frac{1}{\langle T_{\textrm{attach}} \rangle } \approx 0.0025~\textrm{min}^{-1}$. No measurable differences in attachment durations were detected between SIN-treated and untreated T cells (SI Fig. 2).

### Profile likelihood analysis reveals $k_3$ as the driver of SIN-mediated cytotoxic enhancement

With *d* and $k_2$ fixed by direct measurement, the core parameter estimation task focused on the three latent kinetic rates: the conjugation rate $k_1$, the lytic hit delivery rate $k_3$, and the proliferation rate *μ*. We used profile analysis to assess practical identifiability by fixing one parameter at a time while re-optimizing the remaining parameters by minimizing the mean squared errors (MSE) between model predictions and data. This mapped the MSE landscape and enabled the extraction of approximate 95% confidence intervals.

Initial attempts to fit the model to individual datasets resulted in broad, flat valleys in the likelihood profiles for $k_1$ and $k_3$, indicating practical non-identifiability [20]: The data were equally well explained by either a high-binding/low-killing regime or a low-binding/high-killing regime. To systematically investigate this limitation, we performed a simulation-based identifiability analysis using synthetic datasets generated from the model. Profile likelihood analysis [21] of a single synthetic ROI confirmed that the parameters were not practically identifiable under an individual curve fitting strategy (SI Fig. 1). To resolve this ambiguity we adopted a co-fitting strategy, simultaneously fitting the model to multiple synthetic datasets derived from regions of interest (ROIs) with varying initial effector and target cell numbers while maintaining the same initial E: T ratio. This enriched dataset successfully constrained the parameter space, yielding well-defined U-shaped likelihood profiles for both parameters, indicative of practical identifiability (SI Fig. 1). Having established the data requirements for practical identifiability, we applied the co-fitting strategy to the experimental datasets for both SIN-treated and untreated conditions.

#### Parameter comparison (SIN treated vs. untreated):

 

**Fig. 5:**
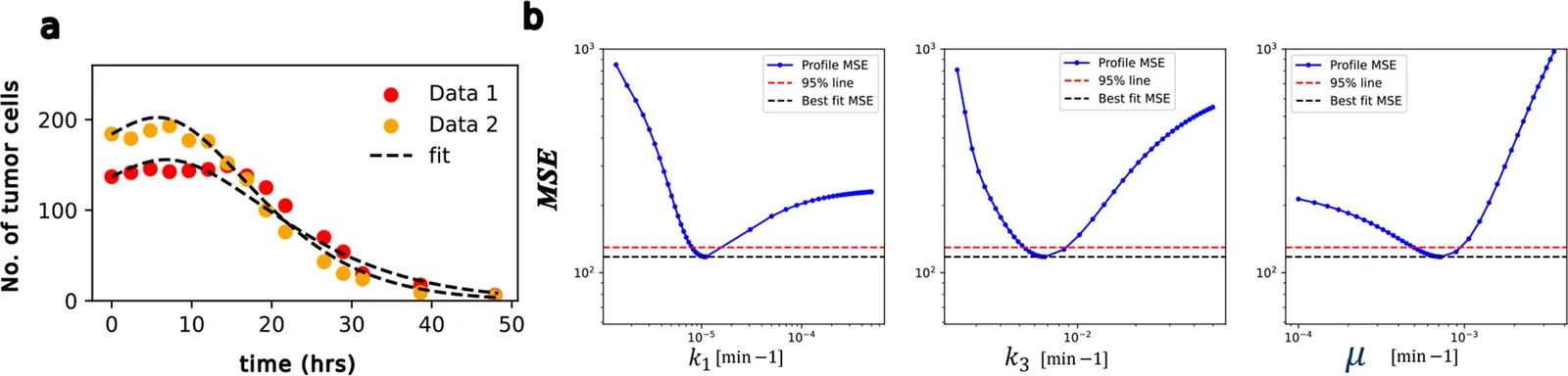
Parameter estimation for the SIN-treated condition: **a** Model fit to tumor-cell count trajectories measured from two independent regions of interest (Data 1 and Data 2) under the SIN-treated condition. The dashed black line shows the model prediction fitted jointly to the ROI-specific trajectories. **b** Profile-MSE curves for the fitted parameters $k_1$, $k_3$, and *μ*, used to assess practical parameter identifiability. The red dashed horizontal line indicates the 95% confidence threshold, and the confidence intervals are determined by the intersections of this threshold with the profile curves

**Fig. 6:**
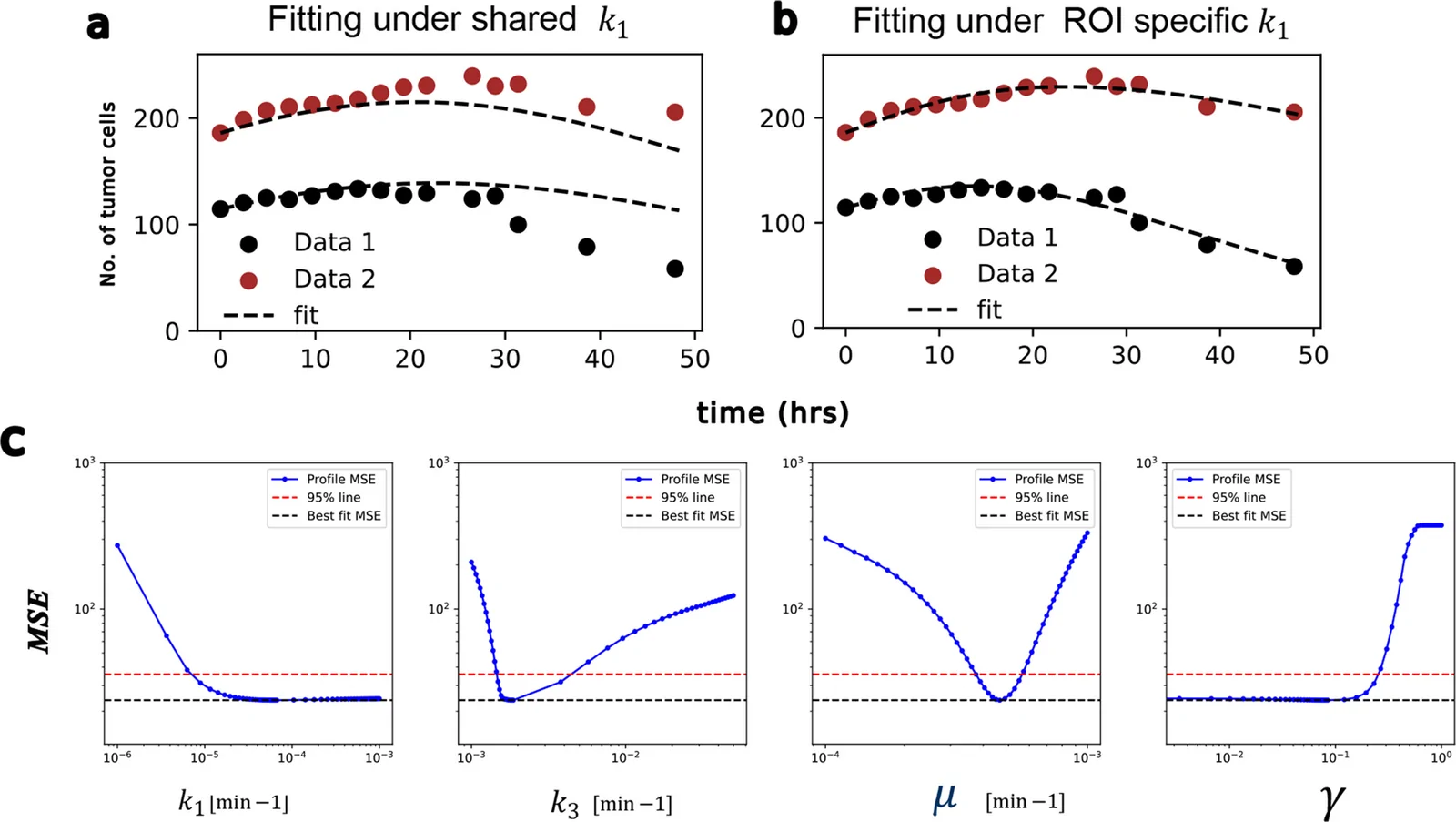
Parameter estimation for the untreated case: **a** Illustration of the model’s inability to adequately fit data collected from different ROIs using a shared set of parameter values, demonstrating poor agreement across datasets. **b** Improved fit achieved by allowing attachment rate $k_1$ to vary between ROIs. **c** Practical identifiability test for the untreated case, showing $k_1$ cannot be uniquely determined with the current dataset

- Conjugation rate ( $k_1$ ): Analysis of the untreated condition revealed unexpected density-dependent effects. In high-density ROIs, untreated T cells appeared less effective at conjugating than predicted by mass action, likely arising from tumor cell clustering or spatial shielding. To account for this, we introduced a phenomenological prefactor *γ* applied to $k_1$, such that the effective conjugation rate in high-density ROIs becomes $\gamma k_1$, correcting for the reduction in available tumor cell surface area due to clustering and spatial shielding. However, the introduction of this additional parameter came at a cost to identifiability: with $k_1$ and *γ* jointly governing the effective conjugation rate, the available data cannot uniquely constrain $k_1$ alone, as the same observed dynamics can be equally well explained by different combinations of $k_1$ and *γ*. This resulted in a flat, unbounded likelihood profile for $k_1$ in the untreated condition, in contrast to the treated condition where no such correction was necessary and $k_1$ was successfully identified (Figs. 5b and 6c). Consequently, a statistically meaningful comparison of $k_1$ between conditions cannot be made, and we conclude that the available data are insufficient to resolve potential differences in conjugation rate.
- Hit Delivery rate ( $k_3$ ): In contrast, the estimated hit delivery rate $k_3$ showed a profound and statistically significant increase in SIN-treated cells, with value approximately 4 times the magnitude of their untreated counterparts (*p = 0.0003*; Table 1).
- Proliferation rate ( *μ* ): No statistically significant difference in *μ* was found between conditions (*p = 0.06*; Table 1), suggesting that SIN exposure does not substantially alter intrinsic tumor growth kinetics.

**Table 1:** Estimated model parameters for SIN-treated and untreated conditions: Mean estimates are reported together with 95% confidence intervals (CI). All parameter values are given in$\textrm{min}^{-1}$. The final column reports the *p*-value for the comparison between SIN-treated and untreated conditions. NA indicates that the upper confidence bound could not be determined from profile likelihood analysis, indicating practical non-identifiability of$k_1$under the current experimental conditions

Parameter	Treated		Untreated		*p*-value
	Mean$(\textrm{min}^{-1})$	95% CI	Mean$(\textrm{min}^{-1})$	95% CI	
$k_1$	$1.1 \times 10^{-5}$	$[0.8,\,2] \times 10^{-5}$	$6.7 \times 10^{-5}$	*NA*	NA
$k_3$	$6.7\times 10^{-3}$	$[5,\,9] \times 10^{-3}$	$1.8 \times 10^{-3}$	$[1.5,\,5] \times 10^{-3}$	0.0003
*μ*	$7.2 \times 10^{-4}$	$[5,\,10] \times 10^{-4}$	$4.62 \times 10^{-4}$	$[3.7,\,5.8] \times 10^{-4}$	0.06

This analysis yields the central finding of the study: the hit delivery rate $k_3$ is approximately four-fold higher in SIN-treated T cells, while conjugate stability ($k_2$) is statistically indistinguishable between conditions. We cannot make an equivalent comparative statement about encounter rate $k_1$, because it remains practically non-identifiable for the untreated condition under the current experimental design. Its contribution could be resolved by encounter-rate measurements using single-cell tracking of T cell migration before conjugate formation. We therefore conclude specifically that accelerated lytic hit delivery is a sufficient and statistically supported mechanistic explanation for the enhanced cytotoxicity of SIN-treated T cells.

### Global sensitivity analysis identifies $k_3$ as the primary determinant of cytotoxic efficacy

To validate the functional relevance of these findings, we performed a global sensitivity analysis to identify parameters that most strongly influence cytotoxic efficacy, defined as the cumulative tumor burden over 48 hours (time-integrated tumor population over 48 hours, AUC), where lower AUC values indicate improved tumor control.

We employed two complementary sensitivity metrics:

1. Spearman rank correlation [22]: This analysis assessed the direction and monotonicity of the relationship between each parameter and tumor burden. As expected, both $k_1$ and $k_3$ showed strong negative correlations with tumor AUC (i.e., increased binding or enhanced killing efficiency reduces tumor burden).
2. Sobol indices [23]: To quantify the magnitude of influence, we computed first-order Sobol indices, which decompose the variance of the model output into contributions from individual parameters.

The Sobol analysis revealed a clear hierarchy of influence. The hit delivery rate $k_3$ emerged as the dominant parameter, explaining the largest fraction of the variance in tumor burden. The conjugation rate $k_1$ was the second most influential factor, while the dissociation rate $k_2$ and apoptosis rate *d* had negligible impacts on the outcome variance.

**Fig. 7:**
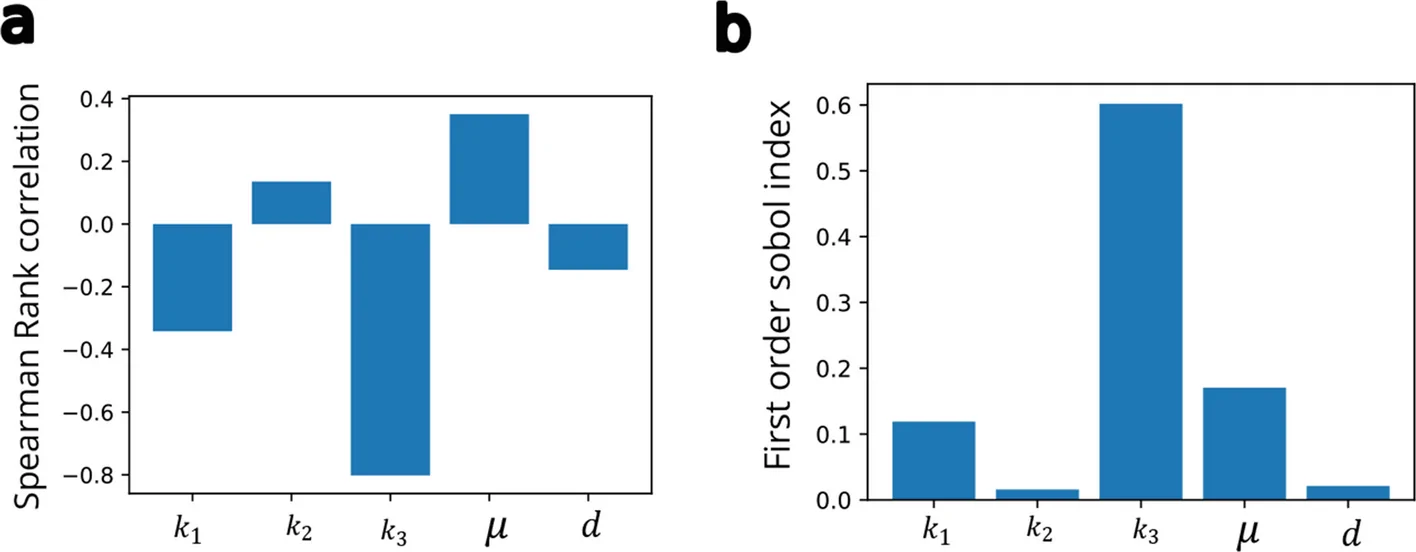
Global sensitivity analysis: **a** Spearman rank correlation coefficients for all parameters, indicating the direction of association with tumor burden. **b** First-order Sobol indices for each parameter, which quantify the contribution of each parameter to the overall variance of the model output, thereby providing insight into the relative importance of each parameter. Sensitivity analysis was performed by varying $k_1$ over $[5.5 \times 10^{-6}, 5.5 \times 10^{-5}]$, $k_2$ and $k_3$ over $[10^{-3}, 10^{-2}]$, *μ* over $[10^{-4}, 10^{-3}]$, and *d* over $[10^{-3}, 10^{-2}]$

This dominance of $k_3$ over $k_1$ can be understood through the lens of the multi-hit bottleneck. In a regime where multiple hits are required (*n=4*) for tumor cell elimination, a T cell that binds frequently (high $k_1$) but delivers hits inefficiently (low $k_3$) remains functionally suboptimal. Consequently, the most effective lever for improving tumor clearance is to maximize the lethality of each contact, rather than the frequency of contacts (Fig. 7).

## Discussion

SIN engineered with the lymphoid cues CCL21 and ICAM-1 enhances the functional potency of adoptively expanded T cells, but the biophysical basis of this improvement has remained unresolved. In particular, it has been unclear whether the enhanced cytotoxicity of CCL21/ICAM-1-treated T cells reflects improved target encounter, greater conjugate stability, or more efficient lethal hit delivery after synapse formation. By integrating long-term live-cell imaging with a mechanistic mean-field model, we were able to separate these alternatives at the level of effective kinetic parameters. Our analysis indicates that CCL21/ICAM-1-based SIN enhances the delivery of lytic hits within the immune synapse. However, its effect on target engagement could not be determined because $k_1$ could not be reliably estimated in untreated T cells.

The estimated increase in the lytic hit-delivery rate, $k_3$, points to an effect of SIN stimulation on processes occurring after immune-synapse formation. A “hit” in our model represents the combined molecular events leading to the delivery of cytotoxic molecules, including Microtubule-Organizing Center (MTOC) polarization toward the synaptic interface, transport of lytic granules along microtubules, granule fusion with the plasma membrane, and diffusion of perforin and granzymes across the synaptic cleft [24]. Previous studies have shown that CCR7 and LFA-1 signalling can enhance synapse-associated signalling and cytoskeletal organization in T cells [25–27]. CCL21 and ICAM-1 stimulation may therefore prime T cells for more efficient lytic execution after synapse formation, a mechanism that we term the *“synaptic lethality hypothesis”*.

Previous work performed under matched culture conditions provides experimental support for the functional improvement predicted by the model. SIN-treated T cells showed improved survival, a shift toward an effector phenotype, reduced expression of proapoptotic and exhaustion-associated genes, and increased expression of the cytotoxicity-associated gene (Gzmm), while granzyme B and perforin expression remained unchanged [9]. These observations are consistent with the model-inferred increase in $k_3$. However, the previous experiments did not measure how rapidly individual T cells delivered lytic hits. Direct validation of $k_3$ would therefore require single-cell comparison of granule polarization, degranulation frequency and timing, CD107a exposure, and cytotoxic-molecule release in SIN-treated and untreated T cells. Simultaneous imaging of degranulation and target-cell caspase activation could additionally test the model-derived estimate that approximately four cytotoxic hits are required to induce target-cell death.

The sensitivity analysis identifying lytic hit delivery rate ($k_3$) as the primary parameter influencing cytotoxic efficacy provides conceptual guidance for T cell engineering strategies. Current approaches often focus on increasing CAR or TCR affinity to enhance tumor recognition ($k_1$). However, our results suggest that such strategies may yield diminishing returns, particularly in the context of solid tumors requiring multiple hits. If $k_3$ is limiting, increasing affinity may instead prolong non-productive T cell–target interactions. Therapeutic optimization should therefore focus on molecular determinants governing $k_3$: This could involve:

- Co-stimulatory Domain Optimization: Selecting CAR intracellular domains (e.g., CD28 vs. 4-1BB) that specifically accelerate granule polarization dynamics [28, 29].
- Granule Loading: Genetic overexpression of Granzyme B or Perforin to ensure that each degranulation event is more potent (effectively increasing the “damage value” of a hit, which in our model is mathematically equivalent to increasing the rate of state transitions) [30, 31].

From a clinical translational perspective, $k_3$ could provide a model-informed functional attribute for comparing adoptive T-cell products. During product development, $k_3$ could be estimated from standardized time-resolved co-culture assays in which T cells are challenged with antigen-positive target cells and the resulting killing dynamics are analysed using the present model. This estimate could subsequently be correlated with faster and more scalable measurements, including CD107a surface exposure, perforin and granzyme B content, to identify surrogate markers suitable for routine potency testing. Rather than serving immediately as a formal release criterion, a product-specific $k_3$ threshold would first need to be established by demonstrating assay reproducibility and an association with functional or clinical outcomes.

While our approach provides rigorous quantitative insights, we acknowledge several limitations. First, the experimental data are derived from 2D in vitro co-cultures. While this simplifies tracking and modeling, it does not fully recapitulate the complex 3D architecture, extracellular matrix barriers, or the immunosuppressive cytokine milieu characteristics of the in vivo tumor microenvironment. Consequently, the conjugation rates estimated here may differ in dense tissue where T-cell motility is physically constrained. Second, our ODE framework assumes a well-mixed population (mean-field approximation). Although appropriate for the suspension-like dynamics of the assay, it ignores spatial heterogeneity and local clustering effects that may influence kill rates in a solid tumor context. While the model identifies that SIN activation enhances the macroscopic killing rate $k_3$, it remains phenomenological regarding the molecular drivers. The specific downstream signaling pathways (e.g., specific upregulation of Perforin/Granzyme B vs. LFA-1 clustering) triggered by CCL21+ICAM1 interaction require further molecular characterization and thereby to establish their connection to the observed cytotoxic phenotype.

## STAR ⋆ methods

### Experimental model and subject details

#### Mice

T-cell receptor (TCR)-transgenic OT-I mice (harboring ovalbumin (OVA)-specific CD8^+^ T cells) were purchased from Jackson Laboratories (Bar Harbor, ME, USA) and bred in the animal facility of the Weizmann Institute. For all experiments, female mice between the ages of 6 and 12 weeks were used. Experiments were performed under protocols approved by the Weizmann Institute’s Institutional Animal Care and Use Committee.

Mice were sacrificed by cervical dislocation performed by trained and licensed animal care personnel. Cervical dislocation is an approved method for euthanasia in mice and is considered to induce immediate loss of consciousness and death when performed correctly. Death was confirmed prior to spleen collection by cessation of respiration and absence of reflexes, including complete flaccidity of the hind limbs. No anaesthetic agents were administered prior to euthanasia, as anaesthetic agents may modulate immune cell phenotypes and function and could therefore affect downstream immunological analyses.

### Method details

#### Substrate functionalization

Substrate functionalization was performed by overnight incubation in phosphate-buffered saline (PBS) containing 10 μg/ml CCL21 and 100 μg/ml ICAM1 (produced by the Structural Proteomics Unit of the Weizmann Institute, Rehovot, Israel) at 37°C.

#### Production and purification of recombinant mCCL21 and mICAM1

Recombinant mouse CCL21 (mCCL21; residues 24-133) containing a C-terminal TEV cleavage site, six-proline rigid linker, and 8×His tag was expressed in Escherichia coli BL21(DE3) cells. The protein was purified by immobilized metal-affinity chromatography followed by C4 reverse-phase HPLC. Recombinant mouse ICAM1 (mICAM1; residues 1-485), containing the same C-terminal TEV cleavage site, six-proline linker, and 8×His tag, was expressed as a secreted protein in Expi293 human cells. The protein was purified from the conditioned medium by immobilized metal-affinity chromatography followed by size-exclusion chromatography. Protein identity and purity were assessed by SDS-PAGE and anti-His immunoblotting. Full production and purification procedures are provided in the SI Note 4.

#### CD8^+^ T cell isolation, activation, and culture

Naïve CD8^+^ T cells were purified (> 95%) from a cell suspension harvested from the crushed spleens of OT-I mice, using a CD8a^+^ T-Cell Isolation Kit and magnetic-associated cell sorting (MACS), according to the manufacturer’s instructions (Miltenyi Biotec, Bergisch Gladbach, Germany). Freshly isolated CD8^+^ T cells were then activated with anti-CD3 and anti-CD28-coated microbeads at 1:1 bead:cell ratio (Miltenyi Biotec) and with 100 U/ml of IL-2 (BioLegend, San Diego, CA, USA). The cells were suspended in RPMI 1640 medium w/o phenol red, supplemented with 10% FBS, 100 U/ml of penicillin, 100 mg/ml of streptomycin, 2 mM glutamine, 1 mM sodium pyruvate, and 50 mM b-mercaptoethanol (Biological Industries, Beit Haemek, Israel) (“complete medium”). The cells were further cultured at 37°C in a 96-well plate (250 μL/well) that was either uncoated or precoated with CCL21-ICAM1 SIN. On day 3 poststimulation, the beads were removed using a magnet, and the contents of all the wells were diluted two fold and subsequently distributed into new wells (coated and uncoated) with fresh medium and IL-2. The cells were then cultured until day 7.

#### In vitro cytotoxic T-cell killing assay

Target B16 cells expressing OVA and GFP (courtesy of Guy Shakhar, Weizmann Institute of Science) were suspended in complete medium. Cells were seeded in a 96-well polystyrene plate (20,000 cells per well) and incubated for 2 hours, to enable their attachment to the substrate. CD8^+^ T cells, activated with CD3/28 microbeads and cultured on ICAM1+CCL21-coated or uncoated surfaces for 7 days, as described above, were subsequently added, on top of the B16 cells, in a ratio of 3:1 (effector: target). The cells were then monitored by time-lapse imaging using a CellDiscoverer 7 microscope (Carl Zeiss Ltd.). Imaging was performed using the TL-oblique contrast method and EGFP channel, using a Plan-Apochromat 20X/0.95 objective and a 1x Tubelens. Illumination was carried out with a TL-LED lamp and LED-Module 470 nm with an excitation wavelength of 488 nm and emission wavelength of 509 nm. Images were acquired using an Axiocam 702 camera (Carl Zeiss Ltd.) at 5 min intervals, for a total period of 48 hours.

#### Cell segmentation and image analysis

For the microscopy-based morphometric analysis of the cytotoxic process, deep learning cell segmentation software, Cellpose [32], was used for semi-automatic identification of target cells. Target cells were segmented based on their morphology, imaged by the TL oblique channel, starting with the built-in ’CPx’ model and refined through a custom model trained via annotation, correction, and validation of the training data. To analyze dynamic morphological changes in target cells during the cytotoxic assay, the trained target cell model was applied to frames captured at multiple time points. Segmentation outputs were subsequently corrected and validated using the GFP channel, which exclusively identifies live target cells. The resulting masks were then processed in Fiji software [33], where a custom macro was used to convert them into ROIs (Regions of Interest) and to overlay these ROIs onto the corresponding GFP channel frames. Finally, various parameters of target cells, including cell area, circularity, and mean GFP intensity, were calculated.

#### Event detection

- Target cell death: Defined as the moment a cell underwent morphological rounding followed by complete loss of cytoplasmic GFP signal. Time of death (the interval between rounding up and complete loss of GFP signal) was recorded for each trajectory.
- Conjugation: T cells (visible in the brightfield channel) were segmented separately. A conjugation event was defined when a T cell and tumor cell were co-localized for ≥ 3 consecutive frames (15 min). Contact duration was recorded to estimate $k_2$.

#### Multi-hit ODE model

We modeled the interaction between T cells and tumor cells using a system of ordinary differential equations (ODEs). The tumor cell population is structured into compartments $C_i$ representing cells that have accumulated *i* lethal hits. The system assumes:

- Tumor growth is logistic and restricted to the undamaged state $C_0$.
- T cell binding ($k_1$) and unbinding ($k_2$) are independent of the damage state of tumor cells.
- Hit delivery ($k_3$) occurs within the complex and transitions the tumor cell to state $C_{i+1}$.
- The final state $C_n$ is apoptotic and decays at rate *d*.

The rate equations for the tumor states $C_i (i = 0 ... n)$ and the complexes are:

1 $$\frac{dC_{i}}{dt} = \underbrace{\mu(C_{i} + [C_{i}T])\left(1 - \frac{C_{\mathrm{total}}}{K}\right)\delta_{i,0}}_{\mathrm{Proliferation}} - \underbrace{k_{1}C_{i}T}_{\mathrm{Binding}} + \underbrace{k_{2}[C_{i}T]}_{\mathrm{Unbinding}}, \quad i = 0, 1, \dots, n-1$$

2 $$\frac{dC_{n}}{dt} = \underbrace{k_{3}[C_{n-1}T]}_{\text{Final Hit}} - \underbrace{d C_{n}}_{\mathrm{Death}}$$

3 $$\frac{d[C_{i}T]}{dt} = \underbrace{k_{1}C_{i}T - k_{2}[C_{i}T]}_{\text{Binding and Unbinding}} + \underbrace{k_{3}([C_{i-1}T] - [C_{i}T])}_{\text{State Transition}}, \quad i = 1, \dots, n-1$$

4 $$\frac{d[C_{0}T]}{dt} = \underbrace{k_{1}C_{0}T - k_{2}[C_{0}T]}_{\text{Binding and Unbinding}} - \underbrace{k_{3}[C_{0}T]}_{\text{State Transition}} ,$$

5 $$\frac{dT}{dt} = -k_{1}T \sum_{i=0}^{n-1} C_{i} + k_{2} \sum_{i=0}^{n-1} [C_{i}T] + k_{3}[C_{n-1}T]$$

6 $$\frac{dC_{\mathrm{total}}}{dt} = \mu(C_{0} + [C_{0}T]) - d C_{n}$$

For the final state *n*, the term −$k_3$ represents the dissociation of the T cell from the dying cell (assuming the T cell leaves to find a new target).

### Quantification and statistical analysis

#### Parameter estimation strategy

To avoid the identifiability problem, we employed a hierarchical estimation approach.

1. Direct Estimation ($d, k_2$): The decay rate d of target cell death was calculated as the inverse of the mean cell death duration ($T_{\mathrm{death}}$) measured from single-cell tracks. The dissociation rate $k_2$ was calculated as the inverse of the mean effector–target attachment duration ($T_{\mathrm{attach}}$), determined by tracking the time interval for which an individual T cell remained attached to a tumor cell.
2. Indirect Estimation ($k_1, k_3, \mu$): The remaining parameters were estimated by fitting the ODE model to the population-level time-course data (Total Tumor Count vs. Time). We used the Trust Region Reflective least-squares algorithm (scipy.optimize.least_squares) to minimize the objective function:

$$J(\theta) = \sum_{t} \left(y_{\mathrm{exp}}(t) - y_{\mathrm{sim}}(t, \theta)\right)^2$$

where *θ* = {$k_1, k_3, \mu$} is the parameter vector. The carrying capacity in the logistic growth model was set to 500, estimated by dividing the total area of the region of interest by the average projected area of an individual tumor cell. 

#### Model selection (AIC)

To determine the optimal number of hits (*n*), we fitted the model for values of *n* ranging from 1 to 9. We compared the models using the Akaike Information Criterion (AIC):

$$AIC = 2k + n_{\mathrm{obs}} \ln(SSR / n_{\mathrm{obs}})$$

where *k* is the number of free parameters, $n_{\mathrm{obs}}$ is the number of data points, and *SSR* is the sum of squared residuals. The value of *n* that minimized the AIC was selected as the consensus model structure (*n = 4*).

#### Profile likelihood analysis

To assess the practical identifiability of $k_1$ and $k_3$, we computed the profile likelihood. For a parameter $\theta_{i}$, the profile likelihood $\chi_{PL}^{2}(\theta_{i})$ is defined as the minimum $\chi^{2}$ value (or weighted SSR) obtainable for the model when $\theta_{i}$ is fixed to a specific value and all other parameters $\theta_{j\neq i}$ are reoptimized. $\chi_{PL}^{2}(\theta_{i}) = \min_{\theta_{j \neq i}} \chi^{2}(\theta)$ A parameter is considered identifiable at the 95% confidence level if its profile likelihood crosses the threshold $\Delta \chi^{2} = 3.84$ (the 95th percentile of the $\chi_{1}^{2}$ distribution) within the biologically relevant range. Flat profiles indicate non-identifiability. We resolved initial non-identifiability by co-fitting multiple datasets with different initial conditions (E:T ratios). For improved visualization, the profile likelihood is presented in terms of mean squared error (MSE). The derivation of the corresponding ΔMSE threshold for the 95% confidence interval is provided in the SI Note 1.

#### Global sensitivity analysis

We performed a variance-based global sensitivity analysis using first-order Sobol indices. As the model output, we considered the area under the curve (AUC) of the tumor population trajectory over 48 hours. The parameter space was sampled using quasi-random Sobol sequences (*N* = 10,000 samples), with parameters varied independently over the following ranges: $k_{1} \in [5.5 \times 10^{-6}, 5.5 \times 10^{-5}]$, $k_{2} \in [10^{-3}, 10^{-2}]$, $k_{3} \in [10^{-3}, 10^{-2}]$, $\mu \in [10^{-4}, 10^{-3}]$, and $d \in [10^{-3}, 10^{-2}]$. We then computed the first-order Sobol index for each parameter, which quantifies its main effect on the variance of the model output while excluding higher-order interaction effects. 

#### Statistical tests

Differences in estimated parameter distributions between SIN-treated and untreated groups were assessed using an approximate Wald-type z-test based on profile-likelihood-derived confidence intervals (SI Note 3).

## Supplementary Information

Supplementary Material 1.

## Data Availability

All raw time-lapse microscopy data and the segmented single-cell tracking datasets generated in this study are available within the paper and its Supplementary Information. This study did not generate new unique reagents. The proteins used in this study were produced by the Structural Proteomics Unit at the Weizmann Institute of Science. • The complete source code for the multi-hit ODE model, including scripts for parameter estimation (profile likelihood) and global sensitivity analysis (Sobol indices), is available on the GitHub page at https://github.com/Mk-guptas/MechanisticModel_Tcell. • Any additional information required to reanalyze the data reported in this paper is available from the lead contact upon request.
