# Supplementary material for: Decoupling T cell cytotoxicity: a CCL21+ICAM1-based synthetic immune niche enhances tumor elimination by accelerating lytic hit delivery
Source: BMC Cancer. 2026 Oct 15;26:1235. doi: 10.1186/s12885-026-16748-y (PMC13670589; doi:10.1186/s12885-026-16748-y)
Supplement: Supplementary file 1 [file 12885_2026_16748_MOESM1_ESM.pdf]

Supplementary information for : Decoupling T Cell Cytotoxicity: A CCL21+ICAM1-Based Synthetic Immune Niche Enhances Tumor Elimination by Accelerating Lytic Hit Delivery

Manish Kumar Gupta, Rawan Zoabi, Sofi Yado, Jörn Starruß, Benjamin Geiger, and Haralampos Hatzikirou

SI Note 1 : Confidence regions from profile statistics

Using the χ^2 profile For a model with *independent Gaussian errors* of (known or pooled) variance σ^2, let

$$\Delta\chi^2(\boldsymbol{\theta}) \;=\; \chi^2(\boldsymbol{\theta}) - \chi^2_{\min}$$

be the likelihood-ratio statistic obtained by fixing the q parameters of interest at $\boldsymbol{\theta}$ and re-optimising all nuisance parameters. Wilks' theorem implies that under the null hypothesis "$\boldsymbol{\theta}$ equals the true value" $\Delta\chi^2$ is asymptotically χ^2_q-distributed. Hence a simultaneous two-sided $100(1-\alpha)\,\%$ confidence region for the q profiled parameters is

$$\mathcal{C}_{1-\alpha} \;=\; \Big\{\boldsymbol{\theta} \;:\; \Delta\chi^2(\boldsymbol{\theta}) < \chi^2_{q,\,1-\alpha}\Big\}. \tag{1}$$

Table 1. 95 % critical values for the difference $\Delta\chi^2$

q profiled parameters	d.f.	$\chi^2_{q,\,0.95}$
1	1	3.84
2	2	5.99
3	3	7.81

Converting the rule to an MSE profile If the vertical axis of the profile plot is the *mean-squared error*

$$\mathrm{MSE}(\boldsymbol{\theta}) \;=\; \frac{\mathrm{SSE}(\boldsymbol{\theta})}{n}, \qquad \mathrm{SSE} = \sum_{i=1}^{n} r_i^2,$$

the link between the two statistics is simply

$$\chi^2(\boldsymbol{\theta}) = \frac{\mathrm{SSE}(\boldsymbol{\theta})}{\sigma^2} = \frac{n\,\mathrm{MSE}(\boldsymbol{\theta})}{\sigma^2}.$$

Therefore the horizontal threshold that encloses the same $100(1-\alpha)\,\%$ confidence region becomes

$$\mathrm{MSE}_{1-\alpha} \;=\; \mathrm{MSE}_{\min} \;+\; \frac{\sigma^2}{n}\,\chi^2_{q,\,1-\alpha}. \tag{2}$$

Computation of threshold To establish the horizontal threshold corresponding to the 95% confidence interval in the MSE profile plots, an estimate of the residual variance σ^2 is required (Eq. 2). Rather than treating the variance as a point-wise quantity, we estimated σ^2 as the mean of the individual per-observation variances computed across all available experimental data points. This pooling approach yields a single, common variance estimate that is more statistically robust than any individual point estimate and is consistent with the assumption

of homoscedastic errors underlying the least-squares framework. The resulting pooled estimate was $\sigma^2 = 100$ (in units of cells^2). Furthermore, since parameter estimation was performed by co-fitting two datasets simultaneously—each comprising 16 time points—the total number of observations entering the objective function was $n = 32$. Substituting these values into Eq. 2, with $q = 1$ degree of freedom (corresponding to the profiling of a single parameter at a time) and the 95th percentile of the χ^2 distribution $\chi^2_{1,\,0.95} = 3.84$, the threshold increment is:

$$\Delta\text{MSE} = \frac{\sigma^2}{n} \cdot \chi^2_{1,\,0.95} = \frac{100}{32} \times 3.84 \approx 12.0 \tag{3}$$

Accordingly, the 95% confidence interval for each parameter is defined as the contiguous set of parameter values for which the profile MSE does not exceed $\text{MSE}_{\text{min}} + 12.0$, where MSE_{min} is the minimum value attained along the profile curve.

SI Note 2: Identifiability test

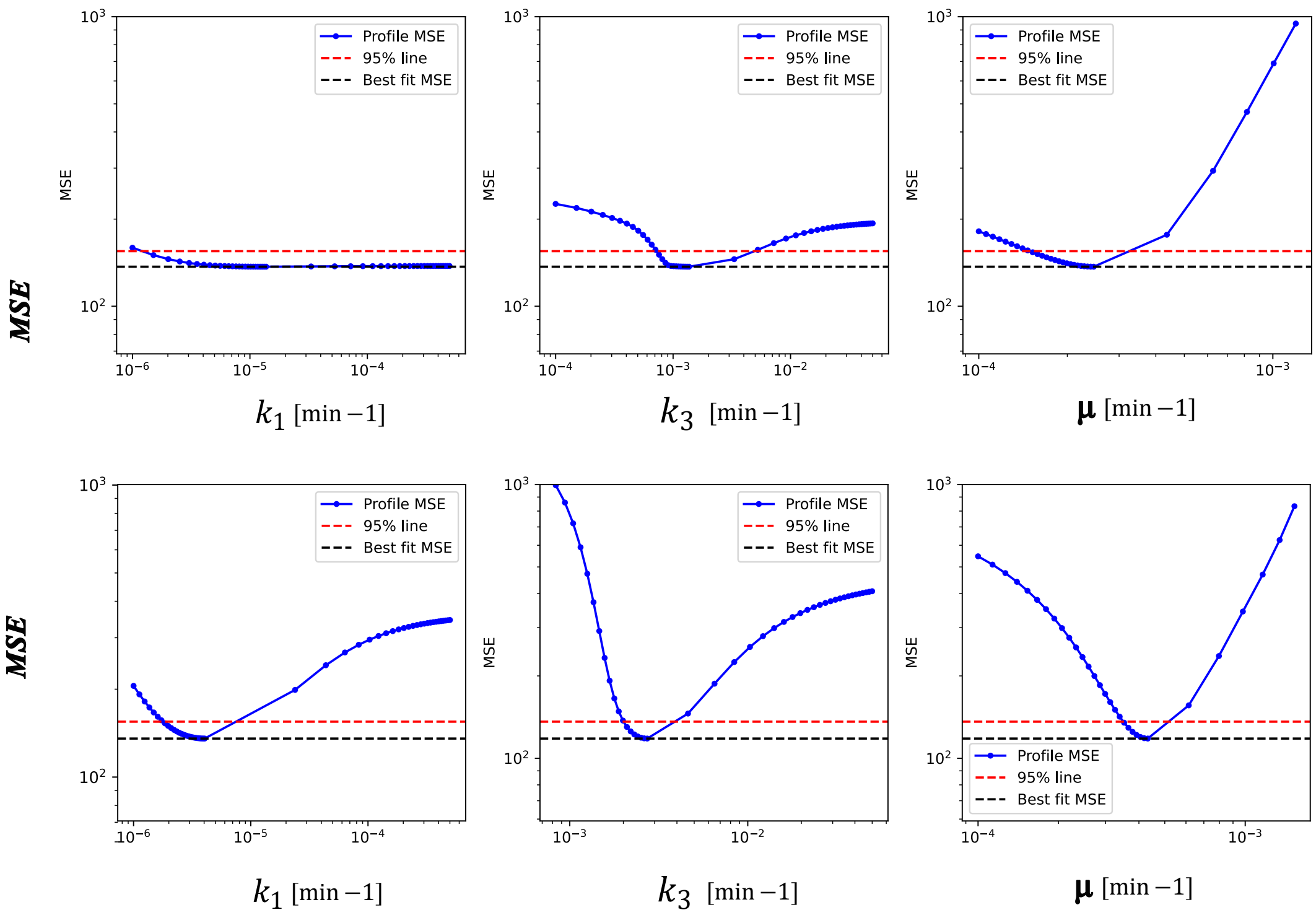

SI Fig. 1. Simulation-based practical identifiability analysis using profile likelihood plots. The upper panels show the analysis performed with a single dataset, where parameters k_1 and k_3 exhibit wide confidence intervals and high standard deviations, indicating poor identifiability. In contrast, the lower panels present the analysis after incorporating an additional dataset, which narrows the confidence intervals and renders k_1 and k_3 identifiable. The solid blue curves represent the sum of squared residuals (SSQ) as each parameter is varied, while the dashed lines denote the best-fit SSQ values.

SI Note 3: Approximate comparison of parameter estimates between SIN-treated and untreated conditions.

To assess whether the fitted parameter values differed between SIN-treated and untreated conditions, we performed an *approximate two-sided Wald z-test* for the null hypothesis

$$H_0: \ \theta_T = \theta_U \qquad (\text{equivalently, } H_0: \ \Delta = \theta_T - \theta_U = 0).$$

Let $\hat{\theta}_T$ and $\hat{\theta}_U$ denote the parameter estimates obtained from the treated and untreated datasets, with corresponding standard errors SE_T and SE_U. Under a large-sample (local normality) approximation, we assume

$$\hat{\theta}_T \approx \mathcal{N}(\theta_T, SE_T^2), \qquad \hat{\theta}_U \approx \mathcal{N}(\theta_U, SE_U^2).$$

Assuming the two estimates are independent, the difference $\hat{\Delta} = \hat{\theta}_T - \hat{\theta}_U$ is approximately normal with

$$\hat{\Delta} \approx \mathcal{N}(\Delta, \ SE_\Delta^2), \qquad SE_\Delta = \sqrt{SE_T^2 + SE_U^2}.$$

We therefore form the Wald statistic

$$z = \frac{\hat{\Delta} - 0}{SE_\Delta} = \frac{\hat{\theta}_T - \hat{\theta}_U}{\sqrt{SE_T^2 + SE_U^2}},$$

which is approximately standard normal under H_0 (i.e., $z \approx \mathcal{N}(0,1)$). The corresponding two-sided p-value is computed as

$$p = 2\left(1 - \Phi(|z|)\right),$$

where $\Phi(\cdot)$ is the cumulative distribution function of the standard normal distribution.

Approximation of standard errors from reported 95% confidence intervals. Because only the estimated means and their 95% confidence intervals were available, the standard errors were approximated from the reported interval widths using the normal approximation

$$SE \approx \frac{\text{upper bound} - \text{lower bound}}{2 \times 1.96} = \frac{\text{upper bound} - \text{lower bound}}{3.92}.$$

This approximation is exact only for symmetric confidence intervals derived from a normal approximation, and should therefore be interpreted cautiously when the reported intervals are asymmetric.

Statistical test for k_3. For k_3, the reported values were

$$\hat{k}_{3,T} = 6.7 \times 10^{-3}, \qquad \hat{k}_{3,U} = 1.8 \times 10^{-3},$$

with 95% confidence intervals

$$[5,\, 9] \times 10^{-3} \qquad \text{and} \qquad [1.5,\, 5] \times 10^{-3},$$

for treated and untreated conditions, respectively. Therefore,

$$SE_T \approx \frac{(9 - 5) \times 10^{-3}}{3.92} = \frac{4.0 \times 10^{-3}}{3.92} \approx 1.020 \times 10^{-3},$$

and

$$SE_U \approx \frac{(5 - 1.5) \times 10^{-3}}{3.92} = \frac{3.5 \times 10^{-3}}{3.92} \approx 8.929 \times 10^{-4}.$$

Thus, the standard error of the difference is

$$SE_\Delta = \sqrt{(1.020 \times 10^{-3})^2 + (8.929 \times 10^{-4})^2} \approx 1.356 \times 10^{-3},$$

and the difference in means is

$$\Delta = 6.7 \times 10^{-3} - 1.8 \times 10^{-3} = 4.9 \times 10^{-3}.$$

Hence,

$$z = \frac{4.9 \times 10^{-3}}{1.356 \times 10^{-3}} \approx 3.614.$$

The corresponding two-sided p-value is

$$p = 2\left(1 - \Phi(3.614)\right) \approx 3.02 \times 10^{-4}.$$

Under this approximation, the difference in k_3 between treated and untreated conditions is statistically significant at the 5% level.

Statistical test for μ. For μ, the reported values were

$$\hat{\mu}_T = 7.2 \times 10^{-4}, \qquad \hat{\mu}_U = 4.62 \times 10^{-4},$$

with 95% confidence intervals

$$[5,\ 10] \times 10^{-4} \qquad \text{and} \qquad [3.7,\ 5.8] \times 10^{-4},$$

for treated and untreated conditions, respectively. Therefore,

$$SE_T \approx \frac{(10 - 5) \times 10^{-4}}{3.92} = \frac{5.0 \times 10^{-4}}{3.92} \approx 1.276 \times 10^{-4},$$

and

$$SE_U \approx \frac{(5.8 - 3.7) \times 10^{-4}}{3.92} = \frac{2.1 \times 10^{-4}}{3.92} \approx 5.357 \times 10^{-5}.$$

Thus,

$$SE_\Delta = \sqrt{(1.276 \times 10^{-4})^2 + (5.357 \times 10^{-5})^2} \approx 1.383 \times 10^{-4},$$

while the difference in means is

$$\Delta = 7.2 \times 10^{-4} - 4.62 \times 10^{-4} = 2.58 \times 10^{-4}.$$

Hence,

$$z = \frac{2.58 \times 10^{-4}}{1.383 \times 10^{-4}} \approx 1.865.$$

The corresponding two-sided p-value is

$$p = 2\,(1 - \Phi(1.865)) \approx 0.0622.$$

Under this approximation, the difference in μ between treated and untreated conditions is not statistically significant at the 5% level.

These p-values should be interpreted as approximate, since they rely on a local Gaussian approximation and on standard errors inferred from reported confidence interval widths. In particular, when confidence intervals are asymmetric or derived from profile likelihood, a likelihood-ratio or profile-based comparison is generally more reliable than the Wald approximation.

SI Note 3: Condition-dependent contact time and death duration

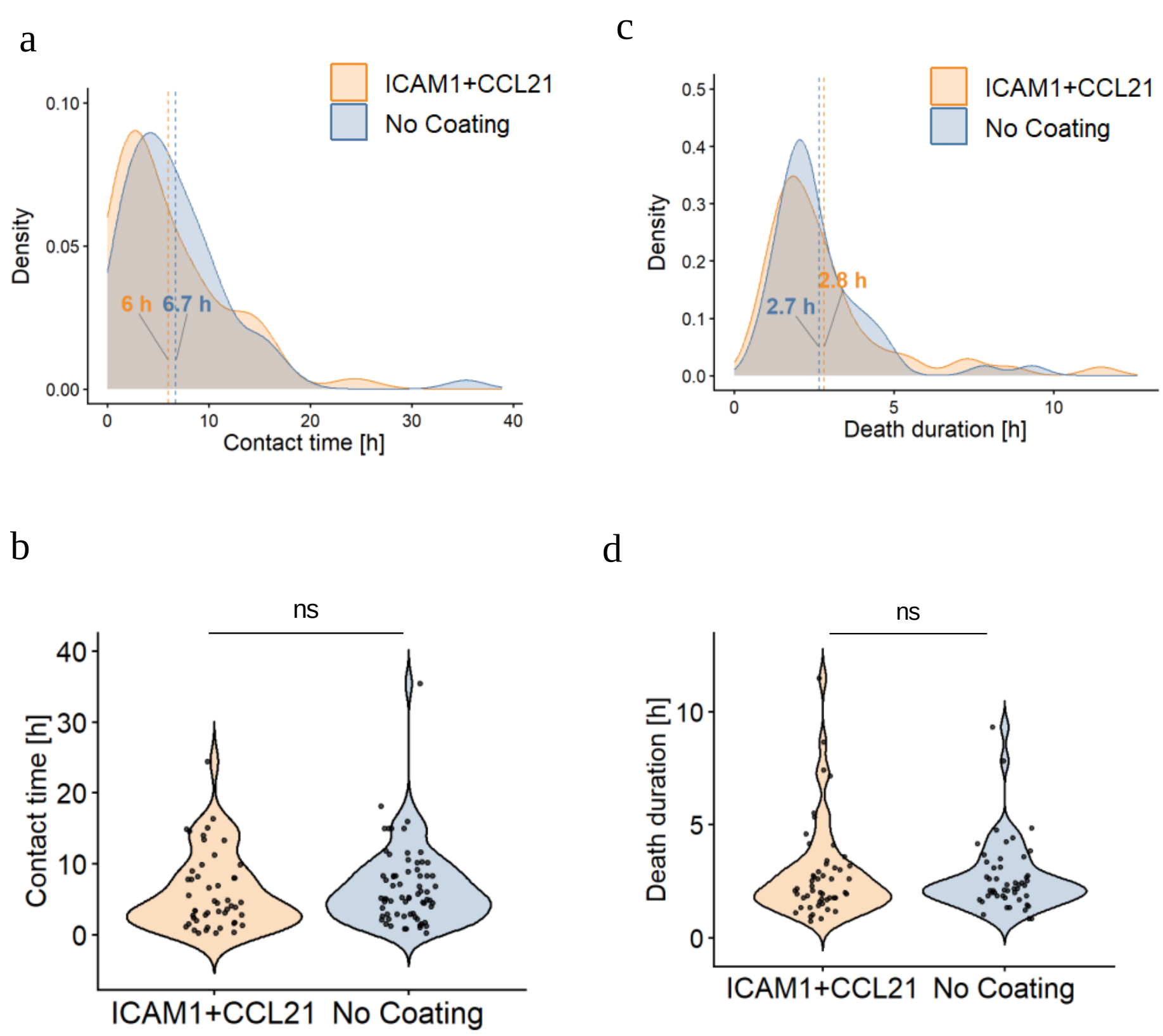

SI Fig 2: Experimental estimate of dissociation rate (k_2) and cell death decay rates (d) for SIN-treated and untreated T cells. (a and b) Density plots of the contact time between target and T cells by pretreatment. The vertical line in **(a)** indicates the mean. Statistical significance was assessed using Welch's t-test ($\alpha = 0.05$; ns, not significant). **(c and d)** Density plots of the interval between target cell rounding and GFP loss ("Cell Death duration") by pretreatment. The vertical line in **(c)** indicates the mean. Statistical significance was assessed using Welch's t-test ($\alpha = 0.05$; ns, not significant).

SI Note 4: Protein production and purification

Production and purification of mouse CCl21 (mCCL21):

The gene encoding mCCL21 (24–133) followed by a TEV cleavage site, a 6 x Proline rigid linker and a C-terminal 8 x His tag was optimized for *E. coli* expression and cloned into pET21a. The resulting construct was transformed into *E. coli* BL21 (DE3) competent cells. Bacterial precultures were grown overnight at 37 °C with shaking and used to inoculate 7.5 L LB. Cultures were grown at 37 °C with shaking to an optical density $OD_{600} = 0.6$. Protein expression was

induced by the addition of isopropyl-β-D-thiogalactopyranoside (IPTG; 200 µM), and the culture was kept at 15 °C with shaking overnight. Cells were harvested by centrifugation, and the pellet was resuspended in 100 ml lysis buffer containing PBS, 150 mM KCl, 10 mM imidazole, 1 mM DTT supplemented with 0.2 mg/ml lysozyme, 20 µg/ml DNase, 1 mM $MgCl_2$, and protease inhibitor cocktail (Calbiochem set 3). After lysis by a cooled protein disrupter (Constant Systems), the soluble fraction was obtained by centrifugation and purified by immobilized metal ion affinity chromatography (IMAC) using a HiTrap FF_5 ml cartridge (Cytiva) using an FPLC system (ÄKTA GE Healthcare Life Sciences) at 4 °C. The column was washed with lysis buffer containing 50 mM imidazole and mCCL21 was eluted in one step with elution buffer (PBS, 150 mM KCl, 500 mM imidazole, 1 mM DTT). The fractions containing mCCL21 were analyzed using SDS-PAGE, pooled and applied to an Xbridge C4 (Waters) reverse phase column operated by an HPLC system (Waters) pre-equilibrated with binding buffer containing 5% acetonitrille, 0.1% TFA in DDW. The protein was eluted with a linear gradient to 95% acetonitrille, 0.1% TFA in DDW. Pure mCCL21 samples from consecutive runs were pooled and lyophilize to dryness. The dry protein was resuspended in PBS and divided into small aliquots before flash freezing in liquid nitrogen. mCCL21 was typically characterized by SDS-PAGE and WB (developed with anti-His).

Expression and purification of mouse ICAM1 (mICAM1): mICAM1 (aa residues 1–485) followed by a TEV recognition site, a 6 x Proline rigid linker and an 8 x His tag was cloned into the pHLSec vector for secretion from Expi293 human cells. Medium (400 ml) containing the secreted protein was collected 72 h post-transfection for purification. After carefully removing the cells, the medium was applied to a Ni column (HisTrap FF_ 5 ml) equilibrated with PBS and the bound mICAM1 was washed with PBS containing 50 mM imidazole and eluted with PBS containing 500 mM imidazole. Fractions containing mICAM1 were pooled, concentrated and injected onto a Superdex_200_16/60 _PrepGrad size exclusion column equilibrated with PBS. The pure mICAM1 migrated at 77 ml as a single peak. The identity of the protein was confirmed by SDS-PAGE developed with Coomassie blue and WB developed with anti-His. Aliquots were flash-frozen with liquid nitrogen and kept at -80 °C.
